# Evolution of knockdown resistance (*kdr*) mutations of *Aedes aegypti* and *Aedes albopictus* in Hainan Island and Leizhou Peninsula, China

**DOI:** 10.3389/fcimb.2023.1265873

**Published:** 2023-09-21

**Authors:** Minghui Zhao, Xin Ran, Dan Xing, Yun Liao, Wei Liu, Yu Bai, Qiang Zhang, Kan Chen, Lan Liu, Mingyu Wu, Zu Ma, Jian Gao, Hengduan Zhang, Tongyan Zhao

**Affiliations:** ^1^ State Key Laboratory of Pathogen and Biosecurity, Beijing Institute of Microbiology and Epidemiology, Beijing, China; ^2^ Jiangxi International Travel Healthcare Center, Nanchang, China; ^3^ Jiangxi Provincial Center for Disease Control and Prevention, Nanchang, China; ^4^ Jiangsu Provincial Center for Disease Control and Prevention, Nanjing, China

**Keywords:** *Ae. aegypti*, *Ae. albopictus*, *kdr*, evolution, population genetics

## Abstract

**Background:**

*Aedes aegypti* and *Aedes albopictus* are important vectors of human arboviruses, transmitting arboviral diseases such as yellow fever, dengue, chikungunya and Zika. These two mosquitoes coexist on Hainan Island and the Leizhou Peninsula in China. Over the past 40 years, the distribution of *Ae. albopictus* has gradually expanded in these areas, while the distribution of *Ae. aegypti* has declined dramatically mainly due to the ecological changes and some other factors such as heavy use of insecticide indoor based on endophagic bloodfeeding of the species.

**Methods:**

This study focused on the knockdown resistance (*kdr*) genes of both mosquitoes, investigated their mutations, and analyzed their haplotype and evolutionary diversity combined with population genetic features based on the ND4/ND5 genes to further elucidate the molecular mechanisms underlying the development of insecticide resistance in both mosquitoes.

**Results:**

Three mutations, S989P, V1016G and F1534C, were found to be present in *Ae. aegypti* populations, and the three mutations occurred synergistically. Multiple mutation types (F1534C/S/L/W) of the F1534 locus are found in *Ae. albopictus* populations, with the three common mutations F1534C, F1534S and F1534L all having multiple independent origins. The F1534W (TTC/TGG) mutation is thought to have evolved from the F1534L (TTC/TTG) mutation. The F1534S (TTC/TCG) mutation has evolved from the F1534S (TTC/TCC) mutation. The most common form of mutation at the F1534 locus found in this study was S1534C, accounting for 20.97%, which may have evolved from the F1534C mutation. In addition, a new non-synonymous mutation M1524I and 28 synonymous mutations were identified in *Ae. albopictus* populations. Correlation analysis showed that the genetic diversity of *Ae. aegypti* and *Ae. albopictus* populations did not correlate with their *kdr* haplotype diversity (P>0.05), but strong gene flow between populations may have contributed to the evolution of the *kdr* gene.

**Conclusion:**

The study of *kdr* gene evolution in the two mosquito species may help to identify the evolutionary trend of insecticide resistance at an early stage and provide a theoretical basis for improving the efficiency of biological vector control and subsequent research into new insecticides.

## Introduction

*Ae. aegypti* and *Ae. albopictus* are vectors of four major arboviruses, dengue, chikungunya, yellow fever and Zika viruses, which have caused severe health burdens and economic losses worldwide. Many years ago, these vectors and viruses were endemic but confined to specific regions; however, in just over 200 years, they have spread to new tropical, subtropical and temperate regions, expanding their global distribution and likely contributing to coordinated outbreaks of arboviral disease epidemics (Kamgang et al., 2017; Leta et al., 2018; Lwande et al., 2020). In the absence of specific drugs or safe and effective vaccines against these viral infections, the control of arboviral diseases relies heavily on integrated mosquito control measures (Kushwah et al., 2020).

Chemical control of vector insects is one of the major initiatives of integrated control, using five main types of insecticides, including organochlorines, organophosphates, carbamates, pyrethroids and insect growth regulators (Li and Zhong, 2008). Pyrethroids have far outstripped the use of organophosphate and carbamate insecticides with their low toxicity, high efficacy and environmental friendliness. However, the long-term and widespread use of these insecticides has led to the intensification of mosquito resistance (Zhou et al., 2019; Li et al., 2021; Egid et al., 2022). As early as the 1980s, *Ae. aegypti* and *Ae. albopictus* in China developed high levels of resistance to insecticides (Wu and Chen, 1988). With frequent iterations and widespread use of insecticides, resistance to insecticides has since developed more rapidly in both species. In addition, *Ae. aegypti* and *Ae. albopictus* have different habitat preferences. *Ae. aegypti* is a domesticated mosquito species that prefers to biting and rest indoors, while *Ae. albopictus* is a semi-domesticated mosquito species that feeds mostly outdoors, so insecticides or electro-thermal repellents commonly used in homes will have a greater effect on *Ae. aegypti* than on *Ae. albopictus*. For example, a survey of insecticide resistance in *Ae. aegypti* and *Ae. albopictus* in Yunnan Province from 2021 to 2023 found that both mosquitoes had developed resistance to permethrin, deltamethrin and cyhalothrin, with *Ae. aegypti* populations in Jinghong have a resistance 825.61 times greater to cyhalothrin. This is also related to the widespread use of insecticide space spraying during the dengue epidemic (Yang et al., 2021; Zheng et al., 2022; Yang et al., 2023). High levels of resistance to pyrethroid insecticides of *Ae. albopictus* have also been reported in 13 dengue-endemic provinces in China (Zhao et al., 2022). High levels of resistance to pyrethroid insecticides all point to the need for rotation (Hayd et al., 2020).

Knockdown resistance (*kdr*) is a form of mosquito resistance to pyrethroid insecticides, mainly due to changes in one or more amino acids in the mosquito’s voltage-gated sodium channel (VGSC), which leads to a change in the conformation of the VGSC, reducing the ability of the pyrethroid molecule to bind as efficiently and further avoiding the toxic effects of the insecticide (Dong et al., 2014; Kamgang et al., 2020). Several *kdr* gene mutations in VGSC structural domains I-IV have been identified in *Ae. aegypti* and *Ae. albopictus*. These include the V410L mutation in domain I; the G923V, L982W, S989P, A1007G, I1011M/V and V1016G/I mutations in domain II; the T1520I, I1532T and F1534C/S/L/W/R mutations in domain III; and the D1763Y mutation in domain IV (Auteri et al., 2018; Gan et al., 2021; Akhir et al., 2022). Most of these mutations have been reported to be closely associated with the development of pyrethroid resistance (Kasai et al., 2011; Moyes et al., 2017; Kasai et al., 2019; Rahman et al., 2021b). In particular, the F1534C mutation form was reported to have first appeared in the *Aedes* mosquito to adapt to the selective pressure of pyrethroid insecticides, and also to provide a platform for the subsequent generation of other mutations, which in turn produced a broader spectrum of pyrethroid resistance (Chen et al., 2019). In addition, the synergistic occurrence of multiple mutations was found to significantly increase resistance in mosquitoes (Smith et al., 2016; Chen et al., 2020; Djiappi-Tchamen et al., 2021). For example, the V410L+V1016I+F1534C mutation in *Ae. aegypti* significantly increased resistance to pyrethroids (Saavedra-Rodriguez et al., 2018). The synergistic mutation V410L+F1534C was significantly more resistant to permethrin than the V410L mutation alone, and the synergistic mutation S989P+V1016G+F1534C was significantly more resistant to deltamethrin and permethrin than the S989P mutation alone (Hirata et al., 2014). S989P + V1016G and V1016G + F1534C synergistic mutations were also found in *Ae. aegypti* populations in China (Li et al., 2015; Lan et al., 2020), and have confirmed that the S989P + V1016G mutation is closely associated with pyrethroid resistance (Chung et al., 2019).

The increasing number of reports of resistance to these two mosquito insecticides worldwide has prompted a major effort by the scientific community to identify the key resistance mechanisms and respond to potential public health emergencies (Auteri et al., 2018). For example, the F1534C mutation in *Ae. aegypti* has been shown to have at least two independent origins, suggesting that selection pressure from DDT and pyrethroid insecticides is responsible for the origin of the F1534C mutation (Cosme et al., 2020). The V410L+F1534C allele mutation may have been caused by the addition of the V410L mutation to the F1534C allele or by a crossover event; the V410L+V1016I+F1534C allele mutation was formed by one or two mutations or recombination steps in the context of the F1534C mutation (Saavedra-Rodriguez et al., 2018; Fan et al., 2020). The S989P+V1016G+F1534C mutant form may be a further mutation type of S989P+V1016G in addition to the V1016G mutation, culminating in a combined form of three mutations (Scott, 2019). It has also been suggested that population migration and hybridization may play an important role in the transmission and maintenance of insecticide resistance alleles in *Ae. aegypti* and *Ae. albopictus*, influencing the generation and further evolution of mutations in the *kdr* gene (Chen, 2019; Rahman et al., 2021a). Studying mutations closely related to *kdr*, analyzing their mutation frequency and haplotyping and the factors influencing their evolution can provide a comprehensive understanding of the distribution and evolutionary pattern of *kdr* mutations, help to identify the development trend of insecticide resistance at an early stage and provide a theoretical basis for subsequent research on new insecticides (Cosme et al., 2020).

Hainan Island and the Leizhou Peninsula are located at the southernmost tip of mainland China and have a tropical (subtropical) climate with high temperatures and rainfall throughout the year. The natural conditions of high temperature and humidity are conducive to the breeding of vector mosquito including of *Ae. aegypti* and *Ae. albopictus*, and which is an important reason why tropical and subtropical regions are prone to insect-borne infectious diseases (Dong and Tan, 2005). There have been several outbreaks of dengue in these areas, and large-scale spraying during outbreaks is bound to cause the further development of mosquito resistance in the area (Wang et al., 2019; Li et al., 2021). In 1988, these two mosquito species were found not to have developed resistance to pyrethroid insecticides (Wu and Chen, 1988). However, in 1994, studies reported that, *Ae. aegypti* and *Ae. albopictus* on Hainan Island had developed resistance to both permethrin and deltamethrin (Li et al., 1994). In 2005, the resistance of these two *Aedes* species to deltamethrin and cyhalothrin was measured again, and the results showed that *Ae. albopictus* was less resistant to deltamethrin and cyhalothrin, while *Ae. aegypti* was susceptible to these two pyrethroids (Zeng et al., 2005). In 2010, a resistance survey of *Ae. aegypti* on Hainan Island and the Leizhou Peninsula conducted showed all the *Ae. aegypti* populations were resistant to deltamethrin, permethrin and cypermethrin (Meng et al., 2015). In 2015, studies reported high levels of resistance to both cypermethrin and cyhalothrin in *Ae. aegypti* in Hainan Island and the Leizhou Peninsula (Li et al., 2015). In recent years, resistance testing of *Ae. albopictus* populations in these areas has revealed high levels of resistance to pyrethroid insecticides, and mutations such as F1534S, F1534C and F1534 L associated with resistance development have been identified (Chen et al., 2021; Li et al., 2021). However, studies on *Ae. aegypti* resistance and *kdr* gene mutation types in these areas have not been reported in recent years, but pyrethroid resistance in *Ae. aegypti* is a global problem (Hirata et al., 2014). In particular, the evolutionary characteristics and factors influencing the *kdr* genes of these two mosquito species in these areas have not been studied. Therefore, in this study, it was investigated the mutations and evolutionary mechanisms of VGSC domain IIS6 and IIIS6 fragments, which are closely associated with the development of pyrethroid resistance in *Ae. aegypti* and *Ae. albopictus*, important vector mosquitoes in Hainan Island and Leizhou Peninsula, China. The study aims to provide theoretical guidance to improve the efficiency of vector control and to guide the scientific use of insecticides and the development of new insecticides in the region.

## Materials and methods

### Sample collection and DNA extraction

From June to October 2021, the group visited Hainan Island and the Leizhou Peninsula to collect samples of *Ae. aegypti* and *Ae. albopictus*. In order to collect more samples of both mosquito species, areas where the presence of *Ae. aegypti* had been reported were selected for extensive sampling. Five geographic strains of *Ae. aegypti* and twenty-two geographic populations of *Ae. albopictus* were collected. Of these, five geographical populations were sampled for *Ae. albopictus* sympatric with *Ae. aegypti* (**Table 1**). *Aedes* larvae were collected from various types of water containers such as buckets, tanks, foam boxes, plastic containers, etc. and reared to adults. Post-fledging mosquitoes were morphologically identified and individually stored in anhydrous ethanol for subsequent nucleic acid extraction. Nucleic acid extraction was performed using the DNeasy® Blood and Tissue Kit (Kadjer, Germany) according to the instructions and the extracted DNA was stored at -20°C.

**Table 1:** Sampling locations and number of *Aedes* mosquitoes.

Localities	*Ae.aegypti* (N)		Total (N)	*Ae.albopictus* (N)		Total (N)	Longitude/Latitude
	Female	Male		Female	Male		
Leizhou Peninsula							
Wushizhen(WS)	15	18	33	7	6	13	E109°50’36’’/N20°33’13’’
Zhanjiangjichang (ZJJC)				14	11	25	E110°22’03’’/N21°12’56’’
Tiaoshunsushe (TSSS)				12	14	26	E110°24’57’’/N21°17’45’’
Nanyousushe (NYSS)				11	14	25	E110°26’27’’/N21°14’53’’
Anshuzhongxin (ASZX)				12	13	25	E110°23’47”/N21°13’30’’
Hainan Island							
Haiweizhen (HW)	12	16	28	15	15	30	E108°49’06’’/N19°25’19’’
Haitouzhen (HT)	17	14	31	15	15	30	E108°56’47’’/N19°30’24’’
Basuozhen (BS)	11	7	18	12	15	27	E108°40’15’’/N19°08’41’’
Yinggehaizhen (YGH)	16	14	30	15	15	30	E108°41’30’’/N18°30’34’’
Meilanjichang (MLJC)				14	12	26	E110°29’46’’/N19°55’58’’
Xiuyinggang (XYG)				13	15	28	E110°17’01’’/N20°01’19’’
Changliuzhen (CLZ)				15	15	30	E110°12’02’’/N20°00’40’’
Hongyunjiudian (HYJD)				15	15	30	E110°20’19’’/N20°01’38’’
Qinglangang (QLG)				13	12	25	E110°49’51’’/N19°32’59’
Huanqiumatou (HQMT)				13	15	28	E110°49’25’’/N19°33’48’’
Basuogang (BSG)				15	15	30	E108°38’02’’/N19°05’59’’
Shisuoxiaoxue (SSXX)				15	15	30	E108°39’22’’/N19°03’15’’
Wandaguangchang (WDGC)				15	15	30	E108°40’57’’/N19°05’19’’
Fenghuangjichang (FHJC)				14	15	29	E109°23’47’’/N18°17’58’’
Fenghuangdao (FHD)				14	14	28	E109°29’05’’/N18°14’02’’
Luopengcun (LPC)				15	15	30	E109°35’53’’/N18°22’02’’
Jishixueyuan (JSXY)				14	14	28	E109°30’57’’/N18°17’41’’
Total (N)	71	69	140	298	305	603	

### Kdr gene amplification

Mutations closely related to pyrethroid resistance have been found in both II and III of the VGSC domain in various *Ae. aegypti* populations worldwide, with the majority of mutations in *Ae. albopictus* populations occurring in domain III (Auteri et al., 2018; Gan et al., 2021). Therefore, in this study, the domain IIS6 and IIIS6 fragments in the VGSC of *Ae. aegypti* and the domain IIIS6 fragment of *Ae. albopictus* were amplified to determine the mutations in the *kdr* gene of both mosquitoes. The primers for amplification were as follows: (1) The primers for the 585 bp IIS6 fragment of *Ae. aegypti* were named AII-F:5’GGTGGAACTTCACCGACTTC3’ and AII-R:5’ GGACGCAATCTGGCTTGTTA3’ (Hamid et al., 2017); (2) The 395bp IIIS6 fragments were named AIII-F:5’ GTGGGAAAGCAGCCGATTCGC3’ and AIII-R:5’ TGTTGAACCCGATGAACAAC3’ (Cosme et al., 2020); (3) The primers for the 780 bp IIIS6 fragment of *Ae. albopictus* were named BIII-F:5’ GAGAACTCGCCGATGAACTT3’ and BIII-R:5’ GACGACGAAATCGAACAGGT 3’, sequenced with the following primer 5’ TAGCTTTCAGCGGCTTCTTC 3’ (Kasai et al., 2011). The amplification reaction system 25 ul consisted of 12.5 ul of PCR reaction solution (TaKaRa, Japan), 8.5 ul of ddH_2_O (TaKaRa, Japan), 1 ul of forward primer and 1 ul of reverse primer (synthesized by Biotech, concentration 10uM), followed by 2 ul of DNA template. The PCR program consisted of one cycle at 94°C for 5 min pre-denaturation, followed by 35 cycles of amplification (94°C, 30 s; 63/60/59°C(AII/AIII/BIII), 60 s; 72°C, 60 s) and a final extension at 72°C for 5 min (BIORAD PCR instrument, USA). Amplification products were subjected to 2% agarose electrophoresis. Positive amplification samples were sent to BGI for sequencing. Samples containing intron insertion/deletion fragments of *Ae. aegypti* IIS6 were cloned, and positive clones selected for sequencing.

### Mitochondrial ND4/ND5 gene amplification

The ND4 genes of *Ae. aegypti* and ND5 genes of *Ae. albopictus* were amplified for population genetic diversity and dispersal analysis, and the primers were as follows: (1) The 380 bp ND4 primers were named AE-F:5’ GTDYATTTATGATTRCCTAA3’ and AE-R:5’ CTTCGDCTTCCWADWCGTTC3’ (Gao, 2021); (2) the 400bp ND5 primers were named AL-F:5’ TCCTTAGAATAAAATCCCGC3’ and AL-R:5’ GTTTCTGCTTTAGTTCATTCTTC 3’ (Kamgang et al., 2011). The amplification reaction system 25 ul consisted of 12.5 ul of PCR reaction solution (TaKaRa, Japan), 8.5 ul of ddH_2_O (TaKaRa, Japan), 1 ul of forward primer and 1 ul of reverse primer (synthesized by Biotech), followed by 2 ul of DNA template. The PCR program consisted of one cycle at 94°C for a 5 min pre-denaturation, followed by 35 cycles of amplification (94°C, 30 s; 48/56°C (AE/AL), 60 s; 72°C, 60 s) and a final extension at 72°C for 5 min (BIORAD PCR instrument, USA). Amplification products were subjected to 2% agarose electrophoresis. Positive amplification samples were sent to BGI for sequencing.

### Data analysis

To better compare the evolutionary characteristics and differences in the *kdr* genes of *Ae. aegypti* and *Ae. albopictus* in the sympatric areas, it was divided all samples into two large groups for analysis. First, five geographic populations of *Ae. aegypti* and five geographic populations of *Ae. albopictus* that sympatric with them were analyzed as a group; Second, the remaining 17 geographic populations of *Ae. albopictus* were analyzed as a group. All sequences were reviewed in BioEdit software and mutation sites were identified. Sequences were then collated separately in Mega v7 (Kumar et al., 2016). The haplotype diversity index (haplotype number-H, haplotype diversity-Hd, nucleotide diversity-π, average number of nucleotide differences-k) was calculated for both *Aedes* species by DnaSP v6.12.03 (Rozas et al., 2003). The TCS network maps were plotted using Popart v1.7 (Clement et al., 2002) and phylogenetic trees were plotted in Mega v7 software using a maximum likelihood model. In addition, molecular variance (AMOVA) and genetic differentiation Fst and Nm gene flow were calculated in ARLEQUIN v 3.1 (Excoffier and Lischer, 2010). Correlation analysis between *kdr* gene haplotype diversity and genetic diversity was assessed using SPSS Statistics.

## Results

### Sampling results and species identification

A comprehensive survey of potential breeding sites for *Ae. aegypti* on Hainan Island and the Leizhou Peninsula were conducted from June to October 2021. The findings were consistent with previous reports that the distribution of *Ae. aegypti* has been significantly reduced (Xie et al., 2011; Chen et al., 2018; Li et al., 2020). Only five populations of *Ae. aegypti* sympatric with *Ae. albopictus* were collected, one from the Leizhou Peninsula and four from Hainan Island. In contrast, *Ae. albopictus* was collected from all areas surveyed (**Table 1**). Morphological identification of adult mosquitoes was carried out 3 days after fledging. Some studies have reported no significant differences between male and female mosquitoes in *kdr* gene mutations, and genetic evolutionary analyses generally need to include samples from both sexes (Artigas et al., 2021; Yang et al., 2021). Therefore, half of the samples from each geographical population of female and male mosquitoes were selected for subsequent molecular experiments.

### Mutations in kdr gene

**Figure 1:**
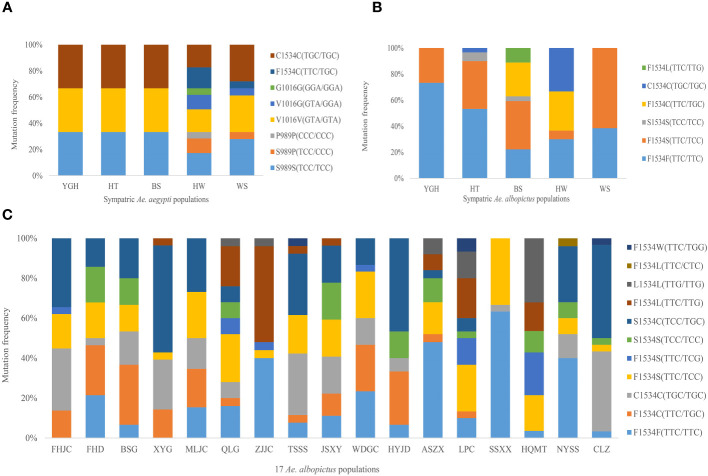
*Kdr* gene mutation frequency of **(A)** Sympatric *Ae. aegypti* populations, **(B)** Sympatric *Ae. albopictus* populations and **(C)** 17 *Ae. albopictus* populations.

A total of three non-synonymous mutations (S989P, V1016G and F1534C) were detected in the *kdr* gene in the five *Ae. aegypti* populations, with the F1534C mutation detected in all populations with 100% mutation frequency, in both heterozygous (F1534C) and homogeneous (C1534C) forms, with the highest mutation frequency in the homogeneous form (86.85%). The S989P and V1016G mutations were synergistic and were only present in the HW and WS populations, with both heterozygous (S989P+V1016G) and homogeneous (P989P+G1016G) forms. Both forms were present in the HW population and only one heterozygous form (S989P+V1016G) was present in the WS population (**Figure 1A**). These mutant forms also occur in association with the F1534C mutation, which was found in only 18 *Aedes aegypti* samples, including 13 in the HW population (S989P/P989P+V1016G/G1016G+F1534C) and 5 in the WS population (S989P+V1016G+F1534C). These 18 samples were sequenced after cloning due to the presence of both of the above mutations, with base insertions or deletions at intron positions. A clonogenic sequence was randomly selected for data analysis. In addition, as the F1534 locus was mutated in all *Ae. aegypti* populations and was the only SNP locus for the IIIS6 fragment. Therefore, subsequent *Ae. aegypti kdr* genotyping studies analyzed only the IIS6 fragment and focused on the S989P and V1016G mutant loci.

Only one non-synonymous mutation (F1534C/S/L) was detected in the *kdr* gene of the five *Ae. albopictus* populations sympatric with *Ae. aegypti*, but the mutation frequencies were significantly different (**Figure 1B**). The F1534S mutation form was detected in all five *Ae. albopictus* populations, whereas the F1534L mutation form was only detected in the BS *Ae. albopictus* population, which had the most diverse mutation types at the F1534 locus (F1534S, F1534C, S1534S and F1534L). The YGH and WS *Ae. albopictus* populations had only one mutation from F1534S.

In addition to the non-synonymous mutation (F1534C/S/L/W) at the F1534 locus, a non-synonymous mutation M1524I (ATG/ATT) was also detected in 17 other *Ae. albopictus* populations. This mutation form was found in the FHJC *Ae. albopictus* population, which occurred in concert with the F1534S mutation. The most common type of mutation at the F1534 locus was S1534C, where there were two base mutations at the same locus, TTC/TTC and TCC/TGC. It was found in all 14 *Ae. albopictus* populations except ZJJC, SSXX and HQMT populations, with a total mutation frequency of 20.97% (99/472). Among them, the LPC *Ae. albopictus* population had the most types of *kdr* gene mutations, with almost all types of mutation forms. In addition, another F1534S mutation (TTC/TCG) was detected in several populations; another F1534L mutation (TTC/CTC) was detected in the NYSS *Ae. albopictus* population; and the F1534W mutation (TTC/TTG) was found in three populations (TSSS, LPC and CLZ). In addition, an additional 28 synonymous mutations were detected in the IIIS6 segment of the *kdr* gene in all *Ae. albopictus* populations. The P1516 locus had the highest mutation frequency at 14.12%, followed by the L1540 and N1541 loci at 10.30% and 10.47%, respectively, and the mutations at the latter two loci occurred synonymously in all populations except one sample; in addition, the F1528 locus also had a high mutation frequency of 9.97%.

### Haplotype diversity of the kdr gene

**Figure 2:**
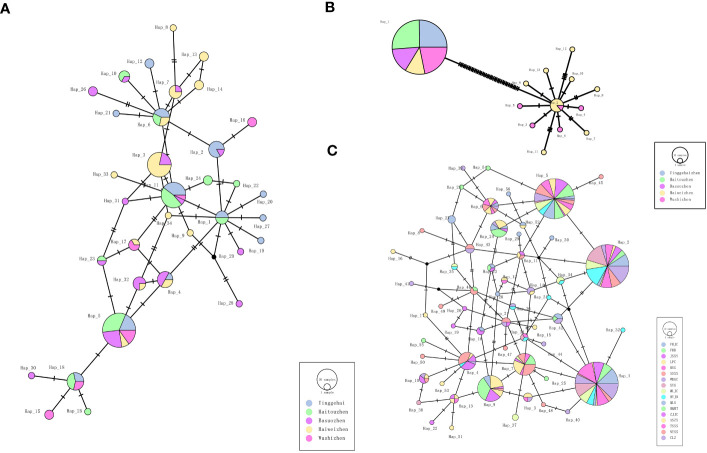
TCS network among haplotypes based on *kdr* gene for **(A)** Sympatric *Ae. albopictus*, **(B)** Sympatric *Ae. aegypti* and **(C)** 17 populations of *Ae. albopictus*. Each line segment represents a single mutation.

The haplotype diversity of the *kdr* gene for the five *Ae. aegypti*, 5 sympatric *Ae. albopictus* and the 17 *Ae. albopictus* populations are displayed in **Table 2**, which showed that the haplotype diversity of the *kdr* gene was low in the *Ae. aegypti* population (Hd=0.000-0.6980, total value 0.2441), but very high in the sympatric *Ae. albopictus* population (Hd= 0.8529-0.9117, total value 0.9224). In addition, the remaining 17 *Ae. albopictus* populations had a lower haplotype diversity than the sympatric *Ae. albopictus* populations (Hd=0.6032-0.9035, total value 0.8726). The *kdr* gene haplotype TCS network showed that the most frequent haplotype in the *Ae*. *aegypti* populations was Hap_1, from which 12 other haplotypes evolved (**Figure 2B**) (Genebank accession number: OR195130). In contrast, the total number of haplotypes in the sympatric *Ae. albopictus* populations was higher (34 haplotypes) and the evolutionary relationships were more complex, with the more common haplotypes being Hap_5, Hap_3 and Hap_11 (**Figure 2A**) (Genebank accession number: OR195129). In addition, the total number of haplotypes in the 17 populations of *Ae. albopictus* was 56, with the most common haplotype being Hap_1, followed by Hap_2 and Hap_5 (**Figure 2C**) (Genebank accession number: OR166252).

**Table 2:** Haplotype diversity of *Ae. aegypti* and *Ae. albopictus* populations.

Mossquito	Population	*kdr*					ND4/ND5				
		N	H^a^	Hd^b^	π^c^	k^d^	N	H^a^	Hd^b^	π^c^	k^d^
Sympatric *Ae.aegypti*	YGH	30	1	0.0000	0.0000	0.0000	30	2	0.3701	0.0011	0.3701
	HT	31	1	0.0000	0.0000	0.0000	31	2	0.4903	0.0015	0.4903
	BS	18	1	0.0000	0.0000	0.0000	18	2	0.4706	0.0014	0.4706
	HW	27	9	0.6980	0.0439	21.2194	28	3	0.5370	0.0033	1.0661
	WS	31	6	0.3011	0.0231	11.1613	33	3	0.5492	0.0057	1.8561
	Total	137	13	0.2441	0.0181	9.2172	140	4	0.5314	0.0029	0.9520
Sympatric *Ae.albopictus*	YGH	30	12	0.9058	0.0130	2.5793	30	2	0.0667	0.0002	0.0667
	HT	30	10	0.8529	0.0143	2.8460	30	1	0.0000	0.0000	0.0000
	BS	27	14	0.9117	0.0164	3.2707	27	3	0.1453	0.0004	0.1482
	HW	30	13	0.8552	0.0123	2.4529	30	2	0.0667	0.0002	0.0667
	WS	13	6	0.8718	0.0139	2.7692	13	2	0.5128	0.0013	0.5128
	Total	130	34	0.9224	0.0154	3.0550	130	5	0.1324	0.0004	0.1349
*Ae.albopictus*	FHJC	29	8	0.7438	0.0118	2.3399	29	4	0.4606	0.0017	0.6404
	FHD	28	8	0.8175	0.0125	2.4921	28	3	0.3148	0.0010	0.3757
	JSXY	27	7	0.7863	0.0121	2.4160	28	3	0.6482	0.0026	0.9921
	LPC	30	13	0.8920	0.0170	3.3724	30	3	0.5586	0.0026	0.9885
	BSG	30	6	0.7471	0.0112	2.2276	30	4	0.6805	0.0027	1.0299
	SSXX	30	13	0.9012	0.0143	2.8368	30	4	0.5816	0.0026	0.9770
	WDGC	30	14	0.9035	0.0151	3.0092	30	5	0.7287	0.0028	1.0529
	XYG	28	5	0.6032	0.0098	1.9444	28	6	0.5847	0.0027	1.0265
	MLJC	26	7	0.8185	0.0120	2.3877	26	3	0.6431	0.0027	1.0154
	HYJD	30	10	0.7540	0.0109	2.1701	30	5	0.5402	0.0022	0.8414
	QLG	25	11	0.8767	0.0149	2.9600	25	6	0.7233	0.0028	1.0667
	HQMT	28	6	0.7302	0.0137	2.7328	28	4	0.4788	0.0018	0.7011
	ZJJC	25	13	0.8900	0.0122	2.4267	25	3	0.4767	0.0014	0.5333
	ASZX	25	13	0.9033	0.0160	3.1800	25	4	0.2300	0.0006	0.2400
	TSSS	26	8	0.7846	0.0121	2.4092	26	2	0.0769	0.0004	0.1539
	NYSS	25	9	0.8400	0.0134	2.6667	25	4	0.3600	0.0013	0.5133
	CLZ	30	6	0.6391	0.0097	1.9310	30	4	0.4943	0.0018	0.6851
	Total	472	56	0.8726	0.0144	2.8556	473	19	0.6571	0.0029	1.0990

a-H: number of haplotypes; b-Hd: haplotype diversity; c-π: nucleotide diversity; d-k: average number of nucleotide differences.

**Figure 3:**
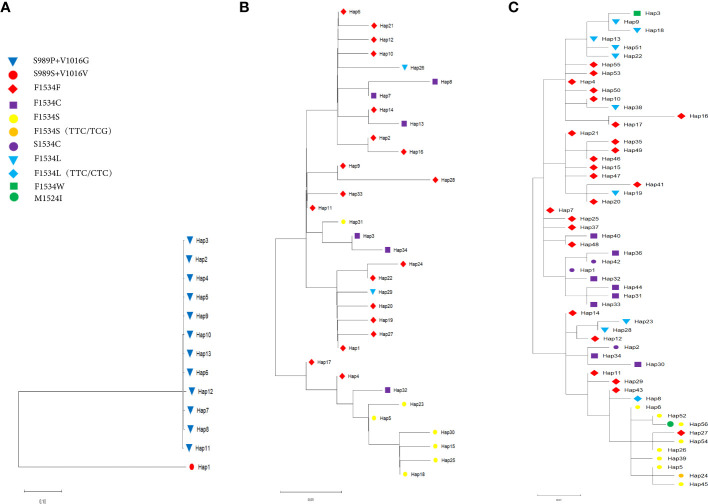
Maximum likelihood tree of the *kdr* haplotypes in **(A)** Sympatric *Ae. aegypti*, **(B)** Sympatric *Ae. albopictus* and **(C)** 17 *Ae. albopictus* populations. ML bootstrap percentages are noted for clades 90%. The different *kdr* mutations are indicated by different symbols.

Phylogenetic tree analysis of these haplotypes and identification of the different *kdr* mutations in the tree (**Figure 3**) showed that the 13 *kdr* haplotypes of *Ae. Aegypti* populations could be divided into two branches, with Hap_1 being the S989S+V1016V wild type with the C1534C homogeneous mutation, while the other 12 haplotypes formed a large branch containing the mutation types S989P+V1016G+F1534C (**Figure 3A**). This also indicated that the S989P+V1016G mutation evolved from the wild-type mutation, which was consistent with the TCS network (**Figure 2B**). The 34 haplotypes of 5 sympatric *Ae. albopictus* populations and the 56 haplotypes of the 17 *Ae. albopictus* populations formed a similar evolutionary pattern, with the wild-type F1534F distributed in all genetic branches (**Figures 3B, C**). **Figure 3B** showed the phylogenetic patterns of five sympatric *Ae. albopictus* populations, from which it could be seen that F1534C, F1534S and F1534L mutations all evolved from the wild type. For example, the F1534S mutant haplotypes Hap_23, Hap_5, Hap_30, Hap_15, Hap_25 and Hap_18 all evolved from the wild haplotype Hap_4; the F1534C mutant haplotypes Hap_3 and Hap_34 all evolved from the wild haplotype Hap_11; the F1534L mutant haplotype Hap_26 evolved from wild haplotype Hap_6, and these results were consistent with the TCS network (**Figure 2A**). **Figure 3C** showed the phylogenetic pattern of the 17 *Ae. albopictus* populations. Similar to **Figure 3B**, the mutation haplotypes all evolved from the wild haplotype, and in addition, several noncommon mutation types could also be seen in the evolutionary direction from the phylogenetic tree and TCS network. For example, the F1534L mutation haplotype (TTC/CTC) Hap_8 evolved from the wild haplotype Hap_43; the F1534S mutation haplotype (TTC/TCG) Hap_24 evolved from the F1534S mutant haplotype (TTC/TCC) Hap_5; F1534W, the only mutation haplotype (TTC/TGG) Hap_3, evolved from the F1534L mutation haplotype (TTC/TTG) Hap_9. In addition, the most common mutation forms S1534C (TCC/TGC) found in this study were all clustered with the F1534C mutation according to the results of phylogenetic tree. For example, S1534C mutation haplotypes Hap_1 and Hap_42 clustered with F1534C mutant haplotypes Hap_31, Hap_32, Hap_33, Hap _36 and Hap_44 clustered together; S1534C mutation haplotype Hap_2 clustered with F1534C mutant haplotypes Hap_34 and Hap_30. In addition, the newly identified M1524I mutation belongs to the Hap_56 haplotype, which could be seen to have evolved from Hap_52 with one base reversal, consistent with the results of the TCS network diagram analysis (**Figure 2C**).

### Population genetic diversity based on ND4/ND5 genes

**Figure 4:**
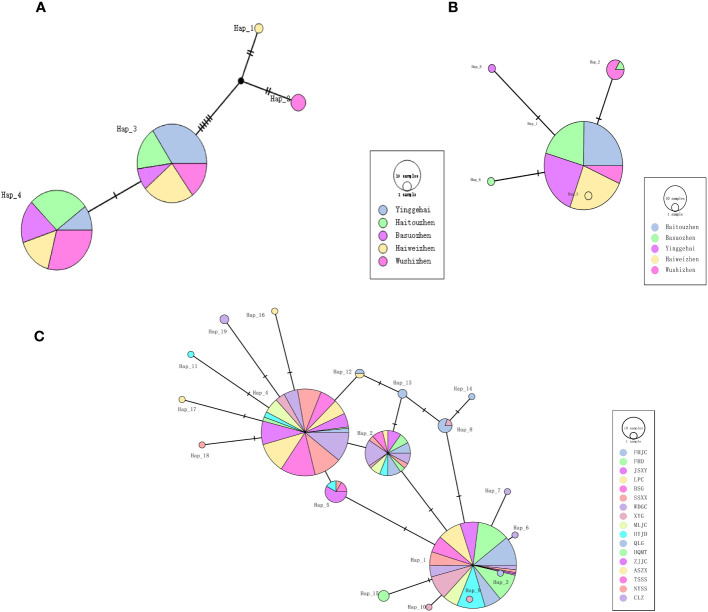
TCS network among haplotypes based on ND4 gene for **(A)** Sympatric *Ae. aegypti*, ND5 gene for **(B)** Sympatric *Ae. albopictus* and **(C)** 17 populations of *Ae. albopictus*. Each line segment represents a single mutation.

**Table 2** shows the haplotype diversity for the ND4 gene of *Ae. aegypti* and the ND5 gene of *Ae. albopictus*. A relatively low haplotype diversity was found both in *Ae. aegypti* (Hd=0.37011-0.54924) and sympatric *Ae. albopictus* populations (Hd=0.00000-0.51282). Four haplotypes and five haplotypes were detected from 140 *Ae. aegypti* and 130 *Ae. albopictus* individuals, respectively (**Figures 4A, B**). The dominant haplotypes of *Ae. aegypti* were Hap_4 and Hap_3, with the other two haplotypes evolving from Hap_3. The dominant haplotype in *Ae. albopictus* was Hap_1, with the other four haplotypes evolving from Hap_1. There was only one haplotype in the HT *Ae. albopictus* population and no intra-population variation. The other 17 *Ae. albopictus* populations had a higher haplotype diversity (Hd=0.07692-0.72874) and 19 haplotypes were detected, with Hap_1 and Hap_4 being the dominant haplotypes and the other haplotypes all evolved from these two haplotypes (**Figure 4C**).

The AMOVA results indicated that the main variation across populations (**Table S1**) was consistent with other studies (Shi et al., 2017; Zhang et al., 2017; Wei et al., 2019; Lv et al., 2020). The results of the interpopulation paired Fst analysis are presented in **Tables S2** and **S3**. **Table S2** showed that the YGH *Ae. aegypti* population was more differentiated from the HT, BS and WS populations with less gene flow, while the HW and WS *Ae. aegypti* populations are less differentiated from the other populations with more gene flow. The WS population of sympatric *Ae. albopictus* showed significant genetic differentiation from the other populations with low gene flow. While other populations of *Ae. albopictus* were less differentiated from each other, with high gene flow. In addition, population differentiation varied among the 17 populations of *Ae. albopictus*, with most populations showing significant genetic differentiation (Fst > 0.05), accounting for 72.26% (101/136) (Guo et al., 2016). However, there were also populations with very low genetic differentiation (Fst < 0) and strong gene flow, accounting for 12.5% (17/136).

To verify whether population genetic diversity affected the evolution of the *kdr* gene, Pearson’s correlation analyses of the genetic diversity and *kdr* gene haplotype diversity was conducted, and found that there was no correlation between these two indices among the *Ae. aegypti* populations (r=0.653, P=0.232>0.05). The same results were also found in populations of sympatric *Ae. albopictus* (r=0.039, P=0.951>0.05) and the 17 *Ae. albopictus* (r=-0.003, P=0.990>0.05).

## Discussion

The dramatic decline of *Ae. aegypti* over the past 40 years was closely linked to vector control efforts in Hainan Island and the Leizhou Peninsula, and the heavy use of insecticides had inevitably led to a reduction in mosquito populations and the development of insecticide resistance (Wang et al., 2015; Li et al., 2021). In the present study, five populations of *Ae. aegypti* and 22 populations of *Ae. albopictus* were collected from different habitats on Hainan Island and Leizhou Peninsula in 2021, focusing on *Ae. aegypti* and *Ae. albopictus* populations in these regions and analyses the mutations and genotyping characteristics of their resistance *kdr* genes, combined with population genetic and dispersal characteristics based on ND4/ND5 genes, to further reveal the differences in the evolutionary mechanisms and factors influencing the development of resistance in the two mosquito species. Detecting specific pyrethroid resistance mutations could help track and map the spread of mosquito resistance, as well as assess the response of mosquito populations to future insecticide interventions to improve the efficiency of mosquito control in Hainan Island and the Leizhou Peninsula (Ishak et al., 2015).

In the present study, only one mutation type at the F1534 locus was found in various populations of *Ae. aegypti*, but its mutation frequency reached 100%, with a frequency of 86.85% for the homozygous form (C1534C). On the other hand, the other two mutations (S989P and V1016G) accompanied the heterozygous form of the F1534 locus mutation (F1534C); no cases of S989P or V1016G alone were found, and the mutation frequency was low (13.14%), which was consistent with the report by Cosme et al. (Cosme et al., 2020). No S989P and V1016G mutations were found in the *Ae. aegypti kdr* gene on the Leizhou Peninsula in 2015, but the F1534C mutation frequency was already 100% (Li et al., 2015). In the present study, it was found that *Ae. aegypti* populations in the Leizhou Peninsula had evolved into S989P and V1016G mutation types, in addition to the 100% F1534C mutation frequency, which may be related to the frequent use of insecticides in these areas in recent years. In addition, the mutation frequency of F1534C in *Ae. aegypti* populations in Hainan Island reported in 2015 was only 87.9% (Li et al., 2015), indicating that after eight years of resistance selection, the F1534C mutation frequency of *Ae. aegypti* in Hainan Island has increased significantly. Some populations have also evolved the S989P and V1016G mutation types (HW). The S989P and V1016G homozygous mutation types were also found in the HW *Ae. aegypti* population. In addition, the HW population had the highest *kdr* haplotype diversity of the *Ae. aegypti* populations, suggesting that the HW *Ae. aegypti* population may have undergone greater insecticide selection. The genetic differentiation data also showed that only the HW *Ae. aegypti* population was less genetically distinct from all four other populations, with a strong gene flow (**Table S2**) (Guo et al., 2016). The strong gene flow may be responsible for the high diversity of *kdr* haplotypes, and also suggests that gene flow between populations may have contributed to the evolution of *kdr* genes (Chen, 2019; Rahman et al., 2021a).

Chung et al. also found a S989P+V1016G mutation pattern in *Ae. aegypti* populations in Taiwan China, and showed their hypothesized evolutionary pathway and closely association with pyrethroid resistance (Chung et al., 2019). Lan et al. found the V1016G+F1534C mutation pattern in *Ae. aegypti* populations in Yunnan province China (Lan et al., 2020). Chen et al. conducted a literature review and found 6 combined mutation patterns in *Ae. aegypti* populations from Lancang-Mekong River Basin, including S989P+V1016G, V1016G+F1534C and S989P+V1016G+ F1534C mutation patterns. S989P+ V1016G+F1534C are mainly found in countries such as Thailand, Myanmar, Cambodia and Laos (Chen and Jiang, 2023), no such mutation pattern has been found in China. It was first found in *Ae. aegypti* populations from China, but it was difficult to infer the evolutionary pattern of the S989P+ V1016G+F1534C mutation pattern in this population because there have been no previous studies of *kdr* gene mutations in *Ae. aegypti* in this region and all S989P+ V1016G+F1534C mutations occurred synergistically. The TCS network and phylogenetic tree of this study could not identify the mechanism by which the S989P and V1016G mutation types arose, and further studies are needed in the future.

In contrast to *Ae. aegypti*, the sympatric *Ae. albopictus* populations had a more complex *kdr* gene evolution, with more mutation types and evolutionary forms in the F1534 locus. In the five *Ae. albopictus* populations, only the F1534 locus had a non-synonymous mutation, but there were three mutation types, F1534C, F1534S and F1534L, with the latter two mutations first identified in *Ae. albopictus* populations in Hainan Island in 2016 (Chen et al., 2016). These mutations, as seen in the TCS network and phylogenetic tree, all evolved from the wild type and underwent multiple independent origins (**Figures 2A**, **3B**). Compared to *Ae. aegypti*, all populations of *Ae. albopictus* have a higher *kdr* haplotype diversity index, with a lower genetic differentiation between populations, except for WS *Ae. albopictus*. In particular, the YGH, TW and HT populations show little genetic differentiation between populations, suggesting strong gene flow between these three populations. The WS population is located on the Leizhou Peninsula and is geographically distant from the other populations, resulting in significant genetic differentiation. To further test whether population genetic diversity affected the evolution of the *kdr* gene, the correlation analyses of two indices (population genetic diversity and the *kdr* gene haplotype diversity for *Aedes aegypti* and *Aedes albopictus*, respectively) was performed. It was found that neither was correlated. The suggested that the high genetic diversity of these two mosquito species did not affect the evolution of the *kdr* genes.

The genetic diversity of the 17 geographic *Ae. albopictus* populations was significantly higher compared to the five sympatric *Ae. albopictus* populations (**Table 2**), suggesting that the presence of *Ae. aegypti* may compete with *Ae. albopictus* for resources and ecological niches, affecting *Ae. albopictus* genetic diversity (Juliano and Lounibos, 2005; Lounibos and Juliano, 2018). However, the present study showed that there was no correlation between the level of genetic diversity and the genotype diversity of the *kdr* gene. In addition to the above three mutation types, F1534S (TTC/TCG), F1534L (TTC/CTC), F1534W (TTC/TGG) and S1534C (TCC/TGC) mutation forms were also found at the F1534 locus. Among them, the F1534S (TTC/TCG) mutation was first identified in the *Ae. albopictus* population in Haikou, Hainan Island, in 2018 (Zhao et al., 2019). This mutation type was found in several populations in Hainan Island and the Leizhou Peninsula in the present study (**Figure 1**). Evolutionary analysis revealed that this mutation evolved from the F1534S (TTC/TCC) mutation type. The F1534L (TTC/CTC) mutation was first identified in the Sanya *Ae. albopictus* population in Hainan in 2021 (Chen et al., 2021). It was found that the mutation occurred in only one population in the Leizhou Peninsula (NYSS) and none in Hainan populations in this study. An evolutionary analysis revealed that this mutation type evolved from the wild type, a similar evolutionary mechanism to the F1534L (TTC/TTG) mutation type. The F1534W mutation was first reported in the Shenzhen population of *Ae. albopictus* in Guangdong Province in 2021 (Chen et al., 2021), but has not yet been reported in *Ae. albopictus* populations in Hainan Island and the Leizhou Peninsula. The F1534W mutation type was found in three populations of TSSS, LPC and CLZ in this study (**Figure 1**). This mutant was found to have evolved from the F1534L (TTC/TTG) mutation based on phylogenetic trees and TCS network analysis, with a further base inversion. The heterozygous S1534C (TCC/TGC) mutation form was first identified in the *Ae. albopictus* population in Haikou in 2018 (Gao et al., 2018), and it was the most common mutation form in this study. Combined with mutational evolutionary analysis, it was clear that this type of mutation was most closely related to the F1534C mutation and was thought to have arisen from further base inversions based on F1534C. There was a fitness cost to the evolution of insecticide resistance in mosquitoes (Kliot and Ghanim, 2012; Deng et al., 2021); the impact of the complex evolutionary form of the F1534 locus in *Ae. albopictus* on its own fitness and vector competence is a very interesting area of research and deserves further study.

The LPC *Ae. albopictus* population had the most diverse mutation types at the F1534 locus. The population was located around county road X820 in Jiyang District, Sanya, which was the only convenient route connecting two major national highways G98 and G224 in Sanya, bridging the urban and rural areas with frequent trade and logistics. The rapid development of trade and logistics, high population mobility and frequent use of insecticides were all factors contributing to the development of resistance in mosquito populations (Sa et al., 2019). The WDGC and ASZX *Ae. albopictus* populations had the highest haplotype diversity in the *kdr* gene, suggesting that these two populations of *Ae. albopictus* have greater variation in the *kdr* gene. Both the WDGC and ASZX *Ae. albopictus* populations were in central urban areas; large-scale annual insecticide spraying in urban areas could significantly increase the selection pressure for resistance in *Ae. albopictus* populations (Xu, 2016; Su, 2019). In addition, there are also highly developed transport and logistics in urban areas. The Fst and Nm values also indicated that the WDGC *Ae. albopictus* population and the other two populations in the city (BSG and SSXX) have low genetic differentiation and strong gene flow, and the ASZX *Ae. albopictus* population has high genetic exchange (Nm>1) with the other three populations in the city (TSSS, NYSS and ZJJC) (**Table S3**). Some studies on the evolution of the *kdr* gene in *Culex quinquefasciatus* suggested that the distribution of *kdr* mutations may be the result of widespread insecticide use and frequent population movements (Zang et al., 2023), and the findings of the present study also supported these conclusions.

It was identified a new non-synonymous M1524I in the FHJC *Ae. albopictus* population. This mutation type has not been previously reported. It had been reported that any non-synonymous mutation on a protein targeted by a chemical insecticide could reduce the affinity of the insecticide for its target protein and lead to resistance (Zeng, 2020). However, as the mutation was only found in one sample, and resistance data for this population were lacking. Therefore, whether this mutation type is associated with insecticide resistance needs to be further investigated. The mutation type T1520I had been reported in *Ae. aegypti* populations at the nearby 1520 locus and occurred in concert with F1534C, but its association with pyrethroid insecticide resistance had not been reported (Kushwah et al., 2015).

In addition to the two non-synonymous mutations detected in *Ae. albopictus* populations, twenty-eight synonymous mutations were also detected, six of which were consistent with studies by Wei and Xu (Xu et al., 2016; Wei et al., 2021). Another study also identified synonymous mutations in VGSC domain III in *Ae. albopictus* populations (Chatterjee et al., 2018; Su, 2019). The presence of these synonymous mutations has increased the genetic diversity of the *kdr* gene in mosquitoes, but their impact on the development of resistance needs to be further investigated.

## Conclusion

This study analyzed the mutations and haplotypes in the *kdr* genes of *Ae. aegypti* and *Ae. albopictus* in Hainan Island and the Leizhou Peninsula, and the influence of population genetic characteristics on them. Three mutations, S989P, V1016G and F1534C, were found in *Ae. aegypti* populations, and the three mutations were first found to occur synergistically in China. Multiple mutation types at the F1534 locus (F1534C/S/L/W) were found in *Ae. albopictus* populations, with the three common mutations F1534C, F1534S and F1534L all having multiple independent origins, and the F1534W mutation likely evolving from the F1534L mutation. The most common F1534 locus mutation found in this study was S1534C, which may have evolved further from the F1534C mutation. In addition, a new non-synonymous mutation M1524I and 28 synonymous mutations were identified in the *Ae. albopictus* population. A comparative analysis with genetic diversity showed that the genetic diversity of *Ae. aegypti* and *Ae. albopictus* populations did not correlate with their *kdr* gene haplotype diversity, but a strong gene flow between populations could contribute to the evolution of the *kdr* gene. The study of *kdr* gene mutations in the two mosquito species can help to identify the development trend of insecticide resistance at an early stage and provide a theoretical basis for improving the efficiency of biological vector control and subsequent research on new insecticides.

## Data availability statement

The original contributions presented in the study are included in the article/**Supplementary Materials**, further inquiries can be directed to the corresponding authors.

## Author contributions

MZ: Conceptualization, Formal Analysis, Investigation, Methodology, Software, Writing – original draft, Writing – review & editing. XR: Formal Analysis, Investigation, Writing – review & editing. DX: Investigation, Resources, Writing – review & editing. YL: Methodology, Writing – review & editing. WL: Resources, Writing – review & editing. YB: Methodology, Writing – review & editing. QZ: Methodology, Writing – review & editing. KC: Resources, Writing – review & editing. LL: Methodology, Writing – review & editing. MW: Resources, Writing – review & editing. ZM: Software, Writing – review & editing. JG: Software, Writing – review & editing. HZ: Conceptualization, Formal Analysis, Resources, Software, Writing – original draft, Writing – review & editing. TZ: Conceptualization, Formal Analysis, Resources, Writing – original draft, Writing – review & editing.
