# Supplementary material for: Evolution of knockdown resistance (kdr) mutations of Aedes aegypti and Aedes albopictus in Hainan Island and Leizhou Peninsula, China
Source: Front Cell Infect Microbiol. 2023 Sep 21;13:1265873. doi: 10.3389/fcimb.2023.1265873 (PMC10552158; doi:10.3389/fcimb.2023.1265873)
Supplement: Supplementary file 1 [file Table_1.docx]

Table S1 AMOVA based on ND4 for *Ae. aegypti* and ND5 gene for *Ae. albopictus*

| Mosquito | Source of variation | df | Sum of squares | Variance components | Percentage of variation (%) | Fst |
| --- | --- | --- | --- | --- | --- | --- |
| Sympatric | Among populations | 4 | 5.353 | 0.03199 | 6.63 |  |
| *Ae. aegypti* | Within populations | 135 | 60.811 | 0.45045 | 93.37 |  |
|  | Total | 139 | 66.164 | 0.48244 | 100 | 0.06630* |
| Sympatric | Among populations | 4 | 1.764 | 0.01507 | 21.36 |  |
| *Ae. albopictus* | Within populations | 125 | 6.936 | 0.05549 | 78.64 |  |
|  | Total | 129 | 8.700 | 0.07056 | 100.00 | 0.21356^*^ |
| *Ae. albopictus* | Among populations | 16 | 84.917 | 0.17706 | 31.64 |  |
|  | Within populations | 456 | 174.440 | 0.38254 | 68.36 |  |
|  | Total | 472 | 259.357 | 0.55960 | 100 | 0.31640^*^ |

Table S2 Fst and Nm matrix calculated ND4 gene for *Ae. aegypti* and ND5 gene for sympatric *Ae. albopictus* populations (Fst values below the diagonal and Nm (Nm=(1/ Fst-1)/4) above the diagonal for every diagonal, bold numbers indicated significant at p<0.05)

| Sympatric *Ae.aegypti* | YGH | HT | BS | HW | WS | Sympatric  *Ae. albopictus* | HT | BS | YGH | HW | WS |
| --- | --- | --- | --- | --- | --- | --- | --- | --- | --- | --- | --- |
| YGH |  | 0.82973 | 0.60002 | 15.63310 | 1.70603 | HT |  | 62.72229 | / | / | 0.26868 |
| HT | **0.23154** |  | / | 5.83569 | 10.388377 | BS | 0.00397 |  | 170.98288 | 170.98288 | 0.61418 |
| BS | **0.29411** | -0.03942 |  | 4.44484 | 32.00806 | YGH | 0.00000 | 0.00146 |  | / | 0.35690 |
| HW | 0.01574 | 0.04108 | 0.05325 |  | 11.16031 | HW | 0.00000 | 0.00146 | 0.00000 |  | 0.35690 |
| WS | **0.12781** | 0.02351 | 0.00775 | 0.02191 |  | WS | **0.48199** | **0.28929** | **0.41193** | **0.41193** |  |

Table S3 Fst and Nm matrix calculated ND5 gene for 17 *Ae. albopictus* populations (Fst values below the diagonal and Nm (Nm=(1/ Fst-1)/4) above the diagonal for every diagonal, bold numbers indicated significant at p<0.05)

|  | FHJC | FHD | JSXY | LPC | BSG | SSXX | WDGC | XYG | MLJC | HYJD | QLG | HQMT | ZJJC | ASZX | TSSS | NYSS | CLZ |
| --- | --- | --- | --- | --- | --- | --- | --- | --- | --- | --- | --- | --- | --- | --- | --- | --- | --- |
| FHJC | 0.0000 | -15.5780 | 1.6438 | 2.5675 | 1.1038 | 0.5427 | 1.0205 | -308.8920 | 1.0977 | -34.5436 | 2.4849 | -24.2885 | 0.1632 | 0.0832 | 0.0828 | 0.1478 | 0.1935 |
| FHD | -0.0163 | 0.0000 | 0.8281 | 1.1512 | 0.6225 | 0.3425 | 0.5974 | 4.8437 | 0.6027 | 5.7916 | 1.7972 | -50.8573 | 0.1033 | 0.0488 | 0.0461 | 0.0932 | 0.1287 |
| JSXY | **0.1320** | **0.2319** | 0.0000 | -10.4833 | -11.0305 | 7.1268 | -16.6220 | 6.0425 | -8.0479 | 3.9902 | 1.2100 | 1.2355 | 0.5468 | 0.2618 | 0.2806 | 0.5424 | 0.7543 |
| LPC | **0.0887** | **0.1784** | -0.0244 | 0.0000 | -18.4982 | 3.9830 | 10.7438 | 56.3111 | -20.6249 | 14.1509 | 1.3761 | 1.8574 | 0.5091 | 0.2309 | 0.2462 | 0.4453 | 0.5904 |
| BSG | **0.1847** | **0.2865** | -0.0232 | -0.0137 | 0.0000 | -44.2641 | 85.0742 | 3.3565 | -7.7688 | 2.4498 | 0.8924 | 0.9210 | 0.8551 | 0.3484 | 0.3846 | 0.7615 | 1.0702 |
| SSXX | **0.3154** | **0.4219** | 0.0339 | 0.0591 | -0.0057 | 0.0000 | 6.2469 | 1.1769 | -203.5020 | 0.9483 | 0.5317 | 0.4883 | 1.8778 | 0.5834 | 0.6864 | 1.6933 | 2.7469 |
| WDGC | **0.1968** | **0.2950** | -0.0153 | 0.0227 | 0.0029 | 0.0385 | 0.0000 | 2.1278 | -13.7416 | 1.6969 | 0.9677 | 0.8100 | 0.5598 | 0.3185 | 0.3453 | 0.6851 | 0.9878 |
| XYG | -0.0008 | 0.0491 | 0.0397 | 0.0044 | 0.0693 | **0.1752** | **0.1051** | 0.0000 | 3.1630 | -8.7621 | 2.8824 | 13.2126 | 0.3136 | 0.1557 | 0.1607 | 0.2703 | 0.3408 |
| MLJC | **0.1855** | **0.2932** | -0.0321 | -0.0123 | -0.0333 | -0.0012 | -0.0185 | **0.0733** | 0.0000 | 2.2984 | 0.9427 | 0.8937 | 0.7260 | 0.3232 | 0.3519 | 0.7316 | 1.0645 |
| HYJD | -0.0073 | 0.0414 | 0.0590 | 0.0174 | **0.0926** | **0.2086** | **0.1284** | -0.0294 | **0.0981** | 0.0000 | 2.1298 | 20.2250 | 0.2602 | 0.1281 | 0.1314 | 0.2243 | 0.2866 |
| QLG | **0.0914** | **0.1221** | **0.1712** | **0.1537** | **0.2188** | **0.3198** | **0.2053** | **0.0798** | **0.2096** | **0.1051** | 0.0000 | 2.0413 | 0.1908 | 0.1133 | 0.1120 | 0.1844 | 0.2278 |
| HQMT | -0.0104 | -0.0049 | **0.1683** | **0.1186** | **0.2135** | **0.3386** | **0.2359** | 0.0186 | **0.2186** | 0.0122 | **0.1091** | 0.0000 | 0.1609 | 0.0823 | 0.0818 | 0.1423 | 0.1828 |
| ZJJC | **0.6050** | **0.7076** | **0.3138** | **0.3293** | **0.2262** | **0.1175** | **0.3087** | **0.4436** | **0.2561** | **0.4900** | **0.5671** | **0.6084** | 0.0000 | 1.0357 | 1.2880 | 2.4840 | 2.2786 |
| ASZX | **0.7502** | **0.8367** | **0.4885** | **0.5198** | **0.4178** | **0.3000** | **0.4397** | **0.6162** | **0.4362** | **0.6612** | **0.6881** | **0.7523** | **0.1944** | 0.0000 | 735.0441 | 4.0360 | 2.1601 |
| TSSS | **0.7513** | **0.8444** | **0.4711** | **0.5038** | **0.3940** | **0.2670** | **0.4200** | **0.6088** | **0.4154** | **0.6555** | **0.6906** | **0.7534** | **0.1626** | 0.0003 | 0.0000 | 16.0049 | 3.6062 |
| NYSS | **0.6285** | **0.7285** | **0.3155** | **0.3595** | **0.2472** | **0.1287** | **0.2674** | **0.4805** | **0.2547** | **0.5271** | **0.5755** | **0.6373** | **0.0914** | 0.0583 | 0.0154 | 0.0000 | -14.5357 |
| CLZ | **0.5637** | **0.6601** | **0.2489** | **0.2975** | **0.1894** | **0.0834** | **0.2020** | **0.4231** | **0.1902** | **0.4659** | **0.5232** | **0.5776** | **0.0989** | **0.1037** | 0.0648 | -0.0175 | 0.0000 |
